# RETRACTED ARTICLE: Topical *Calendula officinalis* L. successfully treated exfoliative cheilitis: a case report

**DOI:** 10.1186/1757-1626-2-9077

**Published:** 2009-11-23

**Authors:** Lúcia Helena Denardi Roveroni-Favaretto, Karina Bortolin Lodi, Janete Dias Almeida

**Affiliations:** Department of Biosciences and Oral Diagnosis, São Paulo State University - UNESP, São José dos Campos, São Paulo, Brazil

**Keywords:** Oral Sucking, Cheilitis, Actinic Cheilitis, Oral Breathing, Angular Cheilitis

## Abstract

Authors describe a case of recurrent exfoliative cheilitis that responded to treatment with a standardized topical preparation of *Calendula officinalis* L. An eighteen-year-old man was referred to UNESP - São Paulo State University, Department of Biosciences and Oral Diagnosis, São José dos Campos Dental School to investigate a chronic dry scaling lesion on his lips. The patient's main chief was aesthetic compromising. Corticoid therapy was suspended and *Calendula officinalis* ointment 10% for *ad libitum* use has been prescribed. The results presented allow the authors to consider *Calendula officinalis* L. as a potential therapy in cases of cheilitis exfoliative.

**Electronic supplementary material:**

The online version of this article (doi:10.1186/1757-1626-2-9077) contains supplementary material, which is available to authorized users.

## Introduction

Exfoliative cheilitis is a reactive process, in which upper, lower or both lips become chronically inflamed, crusted, and sometimes fissured. Dryness of the lips is also an important feature and varying degrees of discomfort can be present. Although exfoliative cheilitis may resolve spontaneously, it often appears periodically and can persist for years [1].

Etiology and pathogenesis are unknown, although some cases may be factitious [1–3]. Chronic lip biting, picking, sucking or unconscious licking of the lips may be the underlying mechanism for trauma and scaling [1]. This entity should be distinguished from contact cheilitis, actinic cheilitis, infectious cheilitis glandularis and granulomatous cheilitis, all conditions affecting the vermilion of the lips, but with distinct ethiopathogenicity. Exfoliative cheilitis may be associated with Candida infection in some cases and may be considered another variant of candidiasis in HIV-positive patients [4].

The difficulties of exfoliative cheilitis therapy are a consensus in literature, authors expose these difficulties through limited results achieved in their cases, treated with conventional therapy, as corticosteroid, cheratolitic agents, antibiotics and sunscreen [1, 5].

In view of the long-term risks of applying steroids and the intractability of the symptoms topical *Calendula officinalis* was selected because of its popularity, relatively low cost and ease of use, administering the ointment daily at home [6–8].

*Calendula officinalis* L. known as calendula or Marigold is an European plant with a bright yellow and orange flower that belongs to Asteraceae family. It's well acclimatized in Brazil, where it is cultivated as an ornamental plant and to produce drugs by pharmaceutical industry [9, 10]. It is a phytotherapic plant rich in biologically active metabolites, like sesquiterpens, alcohol, saponins, triterpens flavonoids, hydroxycoumarin, carotenoids, tannin, and volatile oils (0.1-0.2%) [9, 11, 12]. These components confer antiseptic action, anti-inflammatory, anti-edematous, immunomodulatory activity and antimicrobial effects [10, 13–15].

In dentistry, some of the most common diseases are being treated with great success with phytotherapy. The *Calendula officinalis* L. in this context is indicated to control the bacterial growth into biofilm, against periodontopathogenic bacteria, and oral inflammatory processes that require healing intervention [14, 16].

The aim of this paper is to describe a case of recurrent exfoliative cheilitis successfully treated with topical *Calendula officinalis* L.

## Case presentation

**Figure 1:**
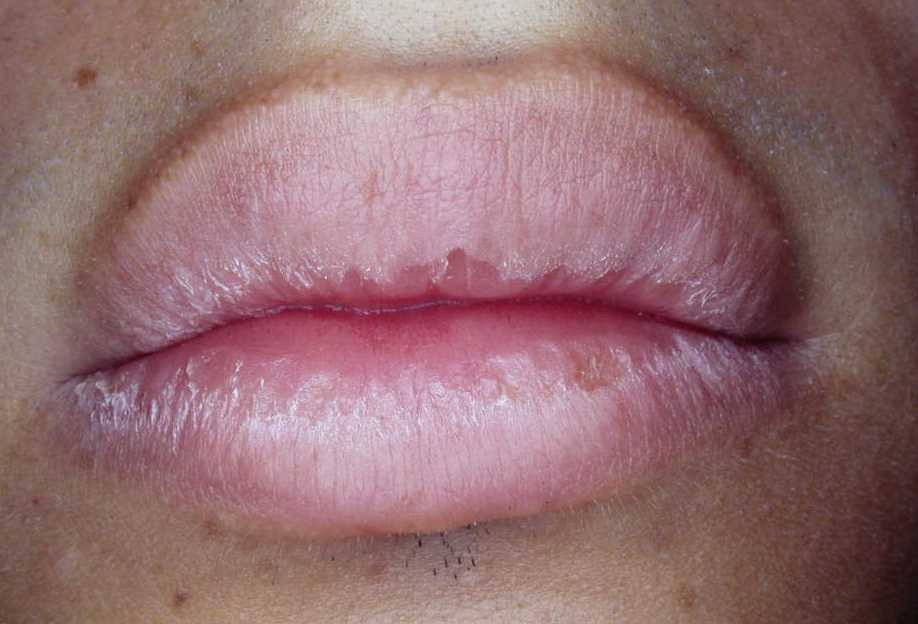
**Dry lips with scaling and crusting particularly involving the vermilion border**.

**Figure 2:**
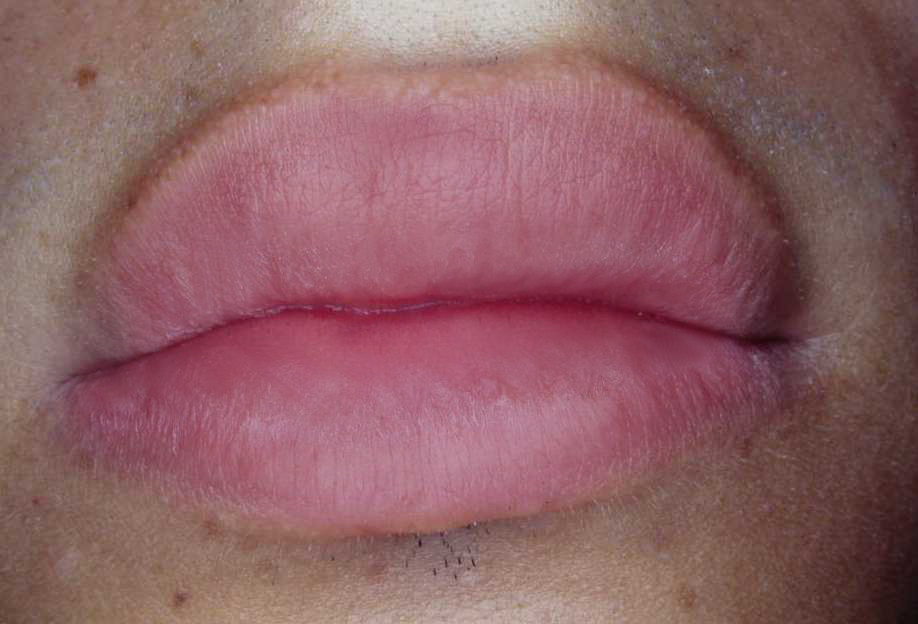
**Lips after treatment with topical** ***Calendula officinalis*** **ointment 10%**.

An 18-year-old Caucasian Brazilian young man was referred to São Paulo State University - UNESP, São José dos Campos Dental School, Department of Biosciences and Oral Diagnosis, São José dos Campos, São Paulo, Brazil to investigate a chronic dry scaling lesion on his lips. The main chief was aesthetic compromising. The patient had consulted several dental practitioners and dermatologists, and they had prescribed 0.1% triamcinolone cream and sunscreen. The symptoms persisted despite the daily use of topic corticosteroid. Cheilitis recurred upon stopping the treatment. The lack of a diagnosis had caused considerable concern to the patient. The patient denied picking his lips. There was no history of any mucocutaneous problem. On examination, he had dry lips with scaling and crusting particularly involving the vermilion border (Figure 1). Oral breathing was evident and considered a contributory factor. A diagnosis of exfoliative cheilitis was made based on the history and the clinical findings. The intraoral examination demonstrated a significant biting line bilaterally into the buccal mucosa. The patient received the support of a speech therapist. In view of the long-term risks of applying corticoids and the intractability of his symptoms it was decided to initiate a treatment with topical *Calendula officinalis* ointment 10%, a preparation done as per Pharmacopoeia (1064), and within seven days just a small area of the lower lip, near the right comissure had a discreet desquamation. After fifteen days the cheilitis had cleared (Figure 2). The patient remains symptom-free and he was advised to use the *Calendula officinalis* ointment 10% when necessary [17].

## Discussion

Exfoliative cheilitis is a benign but often cosmetically unsightly condition. Keratin scales are the main complain and usually it is not painful, though a burning sensation can be present. The etiology is unknown, although self-inflicted injuries and oral habits like oral breathing and oral sucking may initiate the process but not necessarily perpetuate it. When self-inflicted injuries are associated, they are commonly termed "factitious cheilitis". The diagnoses implicate the distinction between other diseases that also affect lips and are generally termed cheilitis. This includes angular cheilitis, plasma cell cheilitis, actinic cheilitis cheilitis glandularis, cheilitis granulomatosa, exfoliative cheilitis and factitious cheilitis [1].

The treatment of exfoliative cheilitis represents a clinical challenge. The response varies from case to case and the criteria for medication choice are empirics. Lesions can spontaneously disappear, but it is common to reappear [3].

The few reported cases in literature describe therapeutics limitations of topic and systemic steroids, antibiotics, keratolytic agents, sunscreen and cryotherapy. Antifungal agents can be administered to patients in whom there is secondary fungal infection but it does not prevent the formation of keratin scales [2]. Medication with anti-depressants was helpful in the case of a 16-year-old male with persistent crusting of the lips with the diagnosis of exfoliative cheilitis [3].

In the case reported, topical *Calendula officinalis* ointment 10% has successfully cleared the condition. The patient's condition was resistant to emollients; only topical steroid helped, but relapsed soon when it was stopped.

Calendula extract heals wounds as well as internal and external ulcers. It is an antiseptic and in addition improves blood flow to the affected area. As an antifungal agent, it can be used to treat athlete's foot, ringworm, and candida infection [8, 13, 18, 19]. The ointment base of *Calendula officinalis* L. is a hidrofobic vehicle most indicated to this therapy because it has good stability, penetrability and is also ease of application [10, 20].

Patients with the diagnosis of exfoliative cheilitis are been indicated to use *Calendula officinalis* ointment 10% with good results. Study showing the results of a larger group of patients will be provided in future.

The results obtained allowed the authors to consider the prescription of *Calendula officinalis* ointment in the treatment of exfoliative cheilitis.

## Consent

Written informed consent was obtained from the patient for publication of this case report and accompanying images. A copy of the written consent is available for review by the Editor-in-Chief of this journal.

## Authors’ original submitted files for images

Below are the links to the authors’ original submitted files for images. Authors’ original file for figure 1 Authors’ original file for figure 2
