# Supplementary figures and images for: RETRACTED ARTICLE: Topical Calendula officinalis L. successfully treated exfoliative cheilitis: a case report
Source: Cases J. 2009 Nov 23;2:9077. doi: 10.1186/1757-1626-2-9077 (PMC2803874; doi:10.1186/1757-1626-2-9077)

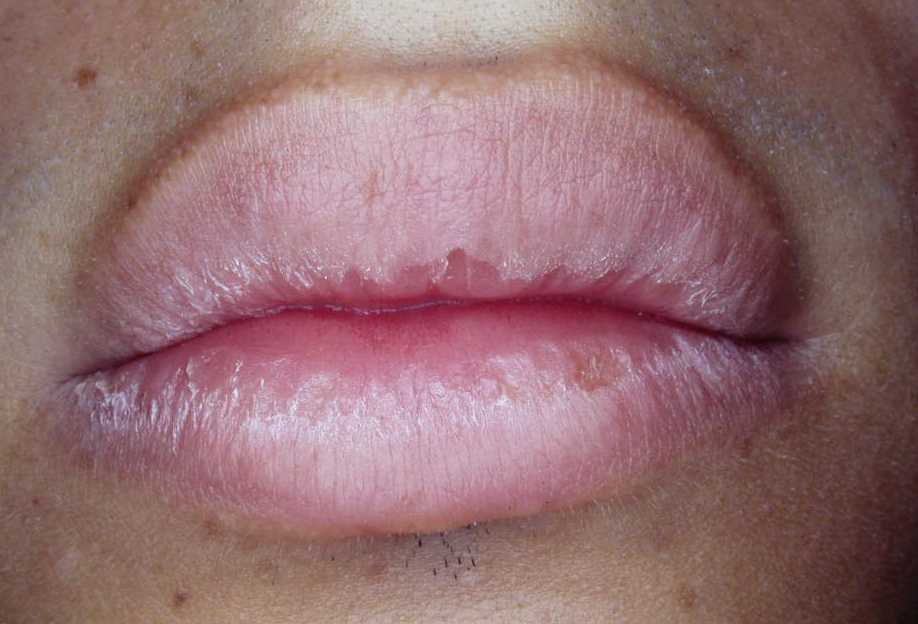

Supplement: Supplementary file 1 — Authors’ original file for figure 1 [file 13257_2009_1282_MOESM1_ESM.tiff]

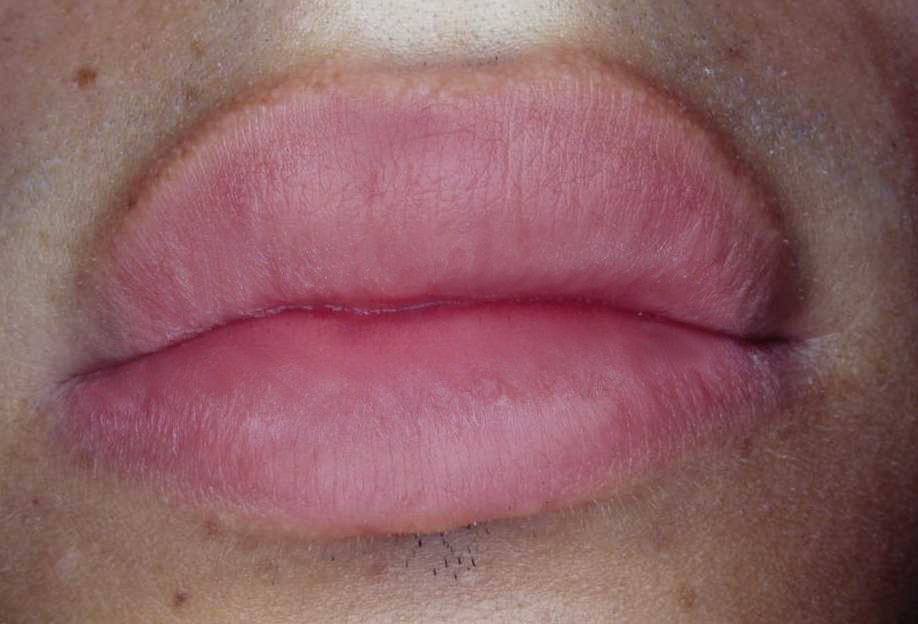

Supplement: Supplementary file 2 — Authors’ original file for figure 2 [file 13257_2009_1282_MOESM2_ESM.jpeg]
